# Propranolol efficiency in prevention of sustained ventricular tachycardia in patients with implanted cardioverter-defibrillator: a case series

**DOI:** 10.3325/cmj.2014.55.75

**Published:** 2014-02

**Authors:** Mislav Puljević, Vedran Velagić, Davor Puljević, Davor Miličić

**Affiliations:** University of Zagreb, School of Medicine, Department for Cardiovascular Diseases, Clinical Hospital Center Zagreb, Zagreb, Croatia

Patients with implanted cardioverter-defibrillator (ICD) often have justified ICD activations no matter if the indication for implantation was primary or secondary prophylaxis of sudden death. First line therapy in the prevention of recidivate ventricular arrhythmia in these patients is antiarrhythmic therapy, but if this is inefficient, arrhythmic substrate radiofrequency (RF) ablation is recommended. Ablation treatment is an accepted procedure in patients with ischemic heart disease, but it has rarely been used in patients with idiopathic and particularly polymorphic ventricular tachycardia (VT). It must be emphasized that acute ablation is relatively successful, but because of substrates progression, relapses of VTs are very frequent (35%) (1). Therefore, antiarrhythmic therapy remains an important therapy after the ICD implantation, before, and often after RF ablation (2).

We present the cases of five patients in whom the prevention of recidivate VTs was achieved only by an old nonselective beta-blocker propranolol (dose 20 × 40 or 2 × 80mg).

Patient 1: a 52 years old woman with ICD implanted as a secondary prevention after out-of-hospital cardiac arrest caused by ventricular fibrillation. There was no structural heart disease, coronarography was normal, and there was only arterial hypertension in patient's history. QTc interval was within the reference range, there were no Brugada syndrome elements, and no sudden death in family history. During the first year after the implantation, ICD was activated for more than 10 times because of polymorphic VT in spite of medicaments therapy. On one occasion even an electrical storm occurred. After the implantation, bisoprolol maximum dose was given, and later in combination with mexiletine, which was stopped as a result of intolerance. Combination with amiodarone was used for a short period of time but with no effects. Only after bisoprolol had been replaced with propranolol, there were no tachycardias and ICD stopped activating. Since then the patient has been followed-up for 5 years.

Patient 2: a 56 years old man with no family or individual heart disease history, or any other serious disease. ICD was implanted as secondary prophylaxis after relapsing syncopes caused by VTs. The first therapy with amiodarone had not prevented multiple ICD activations, so it was replaced by a combination of bisoprolol and mexiletine. Despite the new therapy, ICD continued to activate for several justified occasions (5 times). Only after bisoprolol had been replaced with propranolol, ICD stopped activating. Since then the patient has been followed-up for 3 years. In the meantime, mexiletine therapy has been stopped (it is unavailable in Croatia), so now he takes propranolol only.

Patient 3: a 69 years old man with ICD implanted after relapsing sustained VTs. The patient had no structural heart disease or any signs of ischemia. Coronarography was normal. In spite of the maximum dose of bisoprolol in combination with amiodarone, ICD was reasonably activated on over 20 occasions. On 3 occasions, an electrical storm was detected. Arrhythmia could not have been controlled even by the combination of bisoprolol and mexiletine. Only the replacement of bisoprolol by propranolol led to a complete VT suppression. In the last 3 years, the patient has not had any ICD activation. In the meantime, mexiletine therapy has been stopped.

Patient 4: a 86 years old man. Two years ago, he had inferioposterior myocardial infarction with ST-elevation (STEMI) and a year after he was hospitalized for hemodynamically unstable VT. Slightly reduced ejection fraction (EF 45%-50%) was determined, caused by inferioposterior hypokinesia. Coronarography showed an old collateralized occlusion of the right coronary artery (RCA) and irrelevant changes on other coronary arteries. Sustained VTs repeated daily, despite the use of different antiarrhythmics: bisoprolol, lidocaine, amiodarone, and magnesium. After the temporary electrode had been placed, conversion was achieved by overdrive electrostimulation, but only occasionally. However, electrical cardioversion had to be done in most cases (6 times). Only the combination of propranolol and lidocaine in high doses managed to suppress VT. The patient had a successful RF ablation of VT with propranolol therapy only. During the 2 years of follow-up, he has had no arrhythmia. ICD has not been implanted.

Patient 5: a 60 years old man. After anteroseptal STEMI with severely reduced left ventricular systolic function, the patient developed frequent VTs that responded neither to combination of bisoprolol and lidocaine nor to overdrive stimulation. The use of amiodarone induced a remarkable extension of the QTc interval without suppressing arrhythmia. On most occasions, arrhythmia had to be stopped by electrical cardioversion (5 times). Only after using propranolol instead of bisoprolol, arrhythmia was completely suppressed. When propranolol had been cancelled, because of septic shock, VT relapsed. After the septic shock had been stabilized, ICD was implanted and propranolol was included to the therapy again. Further follow-up (1 year) has not recorded any ICD activation.

In our cases, non-selective propranolol was more effective in suppressing severe VTs than newer selective beta blocker bisoprolol. Unlike bisoprolol, propranolol blocks both beta 1 and beta 2 receptors (20% of beta adrenergic receptors in the heart) and owing to liposolubility also penetrates into the brain (3). Thereby, it can also result in the central inhibition of the sympathetic nervous system. The unique stability effect on the membrane of myocytes has also been described (4). Previous studies showed that propranolol in combination with amiodarone offered the best prevention of VT relapses (5). The reason why propranolol is not widely used in patients with cardiomyopathy is the lack of evidence-based data for these indications. Studies in patients with cardiomyopathy have recently been conducted using only newer beta-blockers (carvedilol, metoprolol, bisoprolol) (6). We believe it could be useful to conduct a study on propranolol, because propranolol possibly has more beneficial effect on the survival of patients with cardiomyopathy than selective beta blockers.
